# Human prenatal exposure to polychlorinated biphenyls (PCBs) and risk behaviors in adolescence

**DOI:** 10.1016/j.envint.2019.04.051

**Published:** 2019-05-27

**Authors:** Aisha S. Dickerson, Yusuf Ransome, Oskar Karlsson

**Affiliations:** a Department of Environmental Health, Harvard T.H. Chan School of Public Health, 665 Huntington Avenue, 1402, Boston, MA 02215, USA; b Department of Social and Behavioral Sciences, Yale School of Public Health, 60 College Street, LEPH 4th Floor, New Haven, CT 06510, USA; c Science for Life Laboratory, Department of Environmental Sciences and Analytical Chemistry, Stockholm University, Stockholm 114 18, Sweden

**Keywords:** Endocrine disrupting chemicals, Alcohol, Smoking, In utero, Polychlorinated biphenyls, Hormesis, Environmental contaminants

## Abstract

Polychlorinated biphenyls (PCBs) are chemicals used in a variety of products before they were widely banned due to toxic effects in humans and wildlife. Because of continued persistence and ubiquity of these contaminants, risk of exposure to people living in industrialized countries is still high. Experimental research show that developmental exposure to PCB may alter function of brain pleasure centers and potentially influence disinhibitory behaviors, including tobacco and alcohol use. Yet, the potential effects of developmental PCB exposure on adolescent substance use have not been studied in humans. We used the Child Health and Development Studies (CHDS), a prospective birth cohort study in the Oakland and East Bay areas of California, to investigate associations between prenatal exposure to PCB congeners (66, 74, 99, 118, 138, 153, 170, 180, 187, and 203) and later disinhibitory behaviors in adolescents, specifically alcohol consumption and smoking, in a randomly selected sample(*n* = 554). Total prenatal PCB exposure was not associated with disinhibitory behaviors, among adolescents. However, the adjusted odds ratio (aOR) for being a current smoker, was higher in subjects within the third quartile of maternal PCB 66 exposure compared to those below the median (aOR = 1.93; 95% CI 1.05, 3.55). The aOR for drinking > 2 alcoholic beverages per week, were also higher for adolescents within the third (aOR = 1.46; 95% CI 0.86, 2.47) and fourth quartile of PCB 66 exposure (aOR = 1.39; 95% CI 0.83, 2.35), but the differences did not reach statistical significance. These results suggest that this specific PCB congener may play a role inducing neurodevelopmental alterations that could potentially increase the risk of becoming a long-term user of tobacco and possibly alcohol. There were no notable differences between magnitude or direction of effect between boys and girls. Future replicate analyses with larger longitudinal samples and animal experimental studies of potential underlying mechanisms are warranted.

## 1. Introduction

Disinhibitory behaviors refers to a collection of conduct problems in adolescents, including tobacco and alcohol use, substance abuse, unsafe sexual activity, and disregard for rule/law. The onset of these risk behaviors shapes adult behavior, and is linked with increased risk of poor educational attainment, future morbidity such as substance use disorders and premature mortality (DuRant et al., 1999; Kipping et ah, 2012; Olsson et ah, 2016). Previous work on the topic have documented that maternal exposures influence offspring externalizing sympto-mology and indicators of disinhibition associated with other childhood disruptive disorders related to impulsivity, hyperactivity, and aggression (Iacono et ah, 2008; Rosenquist et ah, 2017a). Research on the etiology of these outcomes in adolescence has focused on sociodemographic factors, home environment, and cognitive abilities in childhood (Iacono et al., 2008). However, more recent attention has been given to potential biological pathways for these behaviors, including the consideration of prenatal exposures and neurodevelopment of adolescent offspring (Berghuis et al., 2018).

In addition to psychosocial stressors, humans are exposed to a vast array of environmental chemicals that have neurotoxic potential. Chemicals that are classified as persistent organic pollutants (POPs) persist a long time after their introduction into environment. This group of chemicals include polychlorinated biphenyls (PBCs), which are endocrine disrupting chemicals (EDCs) that, despite their widespread ban, remain ubiquitous environmental contaminants due to their chemical stability and lipophilic properties (Kezios et al., 2012; Axelrad et al., 2009). PCBs are comprised of 209 different congeners categorized into 12 dioxin-like compounds and 197 non-dioxin-like PCBs. Non-dioxin-like congeners are more abundant in the environment, with PCBs 28, 52, 101, 138, 153, and 180 being the most prevalent measured in human tissues (Glynn et al., 2000). Until the 1970s, several PCBs were commonly used for industrial processes, but many continue to be used in some developing countries. Due to the sustained persistence and ubiquity of these compounds, risk of exposure to people living in highly industrialized countries is still high (Harrad et al., 2003; Bernard and Fierens, 2002). Unique windows of susceptibility to insults from neurotoxic chemicals occur during gestation and early postnatal development that have no counterpart in the mature brain (Rice and Barone Jr, 2000; Davison and Dobbing, 1968). The ability of PCBs to cross the placenta after maternal exposures, resulting in fetal exposure, is therefore concerning (Vizcaino et al., 2014; Park et al., 2008; Fukata et al., 2005). Animal experimental and epidemiological research indicates that the developing fetus is vulnerable to PCB exposure (Kezios et al., 2012; Allen et al., 1980). In utero exposure to PCBs has been linked to unfavorable birth outcomes such as decreased gestational length and reduce birth weight (Kezios et al., 2012; Govarts et al., 2012; Tang et al., 2018; Tatsuta et al., 2017; Nieuwenhuijsen et al., 2013; Kezios et al., 2017). Developmental PCB exposure has also been reported to result in long-term effects such as increased risk of breast cancer and extended time to pregnancy in daughters (Gennings et al., 2013; Cohn et al., 2012). In addition, PCB exposure has been linked to neurotoxicity and neurological development in children (Roelens et al., 2005; Ribas-Fito et al., 2001; Bell, 2014). For example, PCBs have been associated with altered methylation of genes involved in autism spectrum disorder (Mitchell et al., 2012; Dunaway et al., 2016). Epidemiologic studies have also reported on PCB exposures and reduced attention and impulsivity (Behforooz et al., 2017; Rosenquist et al., 2017b), as well as mood disorders in adults (Gaum et al., 2017).

PCB exposure may alter function of pleasure centers in the brain and potentially influence subsequent inhibitory behaviors. Dopaminergic signaling is a well-known mechanism for decision-making, motivation, and ability to experience pleasure (Bressan and Crippa, 2005). The rewarding and reinforcing effects of drugs involves several endogenous neurotransmitter systems but are believed to be largely mediated by the mesolimbic and mesocortical dopaminergic systems (Koob, 2009). The striatum and prefrontal cortex of the brain are key regions of dopaminergic action involved in reward-seeking behaviors, such as substance abuse and sexual behavior. PCB exposure can disrupt these dopaminergic neural circuitries, by affecting dopamine levels, transporters, and receptors (Bell, 2014; Mariussen and Fonnum, 2001), and could therefore potentially enhance desires for pleasurable experiences (Sharot et al., 2009). It is evident that specific PCB congeners may target different brain regions and/or induce diverse biological responses. For example, developmental exposure to PCB 77 and 126 have been shown to increase dopamine levels in the prefrontal cortex of adolescent and adult rats, while PCB 47 can decrease the dopamine levels in the same region (Seegal et al., 2005). Developmental PCB exposure also induced reduced long-term potentiation (LTP) and decreased the maximal number of N-methyl-D-aspartate (NMDA) receptor binding sites in the cortex of male rats (Altmann et al., 2001). Furthermore, early-life PCB exposure caused behavioral alterations and decreased NMDA receptor binding in the striatum, prefrontal cortex, and hippocampus of female, but not male, adolescent mice (Tian et al., 2011). Other animal studies show that developmental exposure to PCBs induce long-term alterations in the hypothalamus-pituitary-adrenal (HPA) axis (Zimmer et al., 2009; Zimmer, 2012), which is an essential system for stress response regulation that have extensive interactions with the above mentioned neurotransmitter systems (Moghaddam, 2002; Piazza et al., 1996; Rouge-Pont et al., 1998; Lovallo, 2006). Interestingly, a more recent study showed that developmental exposure to PCBs induced higher alcohol consumption in adolescent female rats, suggested to be mediated through increased estrogen levels (Lombardo and Peck, 2018). However, the potential effects of developmental PCB exposure on adolescent substance use outcomes in humans are not well known. The aim of this prospective cohort study was therefore to investigate associations between prenatal exposure to PCBs, and risk behaviors (i.e. alcohol consumption and smoking) at adolescence in a sample randomly selected from Child Health and Development Studies (CHDS) participants, based in the Oakland and East Bay areas of California.

## 2. Methods

### 2.1. Study design and participants

CHDS was a study of prenatal determinants of later health of offspring, per follow-up during infancy, childhood, adolescence, and adulthood (van den Berg et al., 1988). Methods for data collection have been previously described (van den Berg et al., 1988). In brief, pregnant mothers were recruited in the Oakland and East Bay areas of California between 1959 and 1966 if they were participating in the Kaiser Per-manente Health Plan. For the purposes of this study, we used interview responses obtained during pregnancy and at age 15-18 years (n = 554). Demographic characteristics of the included study subjects are presented in Table 1.

### 2.2. Exposure measurement

In utero exposure to PCBs was estimated by analyzing maternal serum samples collected during the early postpartum period, within three days after delivery of the child. After serum fractionation, samples were stored at −20 °C until thawing for analysis in 2007-2008. Serum concentrations of 11 PCB congeners (66, 74, 99, 118, 138, 153, 170, 180, 187, 194 and 203) were measured by mass spectrometry using previously described analytical methods (Rogers et al., 2004; Brock et al., 1996; Wolff et al., 1993; Gammon et al., 2002). For this analysis only lipid-adjusted values were used, which was not provided for PCB 194. Total PCBs is the sum of all of the lipid-adjusted congeners. Limit of detection (LOD) was 0.07 ng/mL for individual compounds based on three times the standard deviation of the levels found in the lowest quality control pool. When the serum pool and blanks were considered together, the LOD was 0.01-0.1 ng/mL. The instrumental LOD based on a peak-to-noise ratio of 3, was 0.01-0.03 ng/mL for tetra- through hepta-chlorobiphenyls, using 1-1.5 mL serum (Cohn et al., 2011).

### 2.3. Behavioral measures

Responses regarding type, amount, and frequency of alcohol use and smoking status were obtained via telephone interviews administered at the age 15-18 by trained research staff. Question topics included, smoking status, ever smoked at least one cigarette per day, number of drinks per week, how often subject drank beer, wine, of other spirits, how often subject was “high or tight” from drinks, and who the subject commonly drank with.

### 2.4. Covariates

Detailed information on maternal and paternal characteristics, including alcohol consumption during pregnancy, smoking status, annual household income, and education level, were obtained from interview responses of the mother during pregnancy. Additionally, other demographic data, such as race and age of the mother and marital status, along with anthropometric measures and other birth outcomes were abstracted from maternal and pediatric medical records.

### 2.5. Statistical analysis

When siblings were identified, the younger child was removed from the analysis to ensure independence of observations (n = 9). Additionally, subjects were removed because no PCB exposure data were available (n = 20) and because no questionnaire was administered (n = 7). Mother’s race and child’s gender and age at the time of the questionnaire, and with whom the child reported drinking were included in the analysis as a priori confounders. We used univariable logistic regression models to identify potential confounding factors, known to be associated with disinhibitory behaviors, associated with maternal serum PCB levels (P < 0.25). Additionally, we tested for effect modification of results by subject sex and found no evidence of effect in stratified analyses. We tested for multicollinearity of included confounders using criteria of correlations above 0.6 and Eigen values close to 0. We found no evidence of multicollinearity among the covariates. We also used logistic regression models to evaluate associations between disinhibitory behaviors (e.g. current smoking status, ever smoked one cigarette per day, drank > 2 alcoholic beverages per week) with maternal lipid-adjusted PCB levels (here in referred to as PCB levels) categorized into quartiles, and exposures below the median serving as the reference measure. To investigate associations between frequency of disinhibitory behaviors and PCB levels, we used negative binomial models. For each analysis, statistical structure (e.g. continuous vs categorical) of PCB exposures included in the model was determined based on fit via Akaike information criterion.

**Fig. 1.**
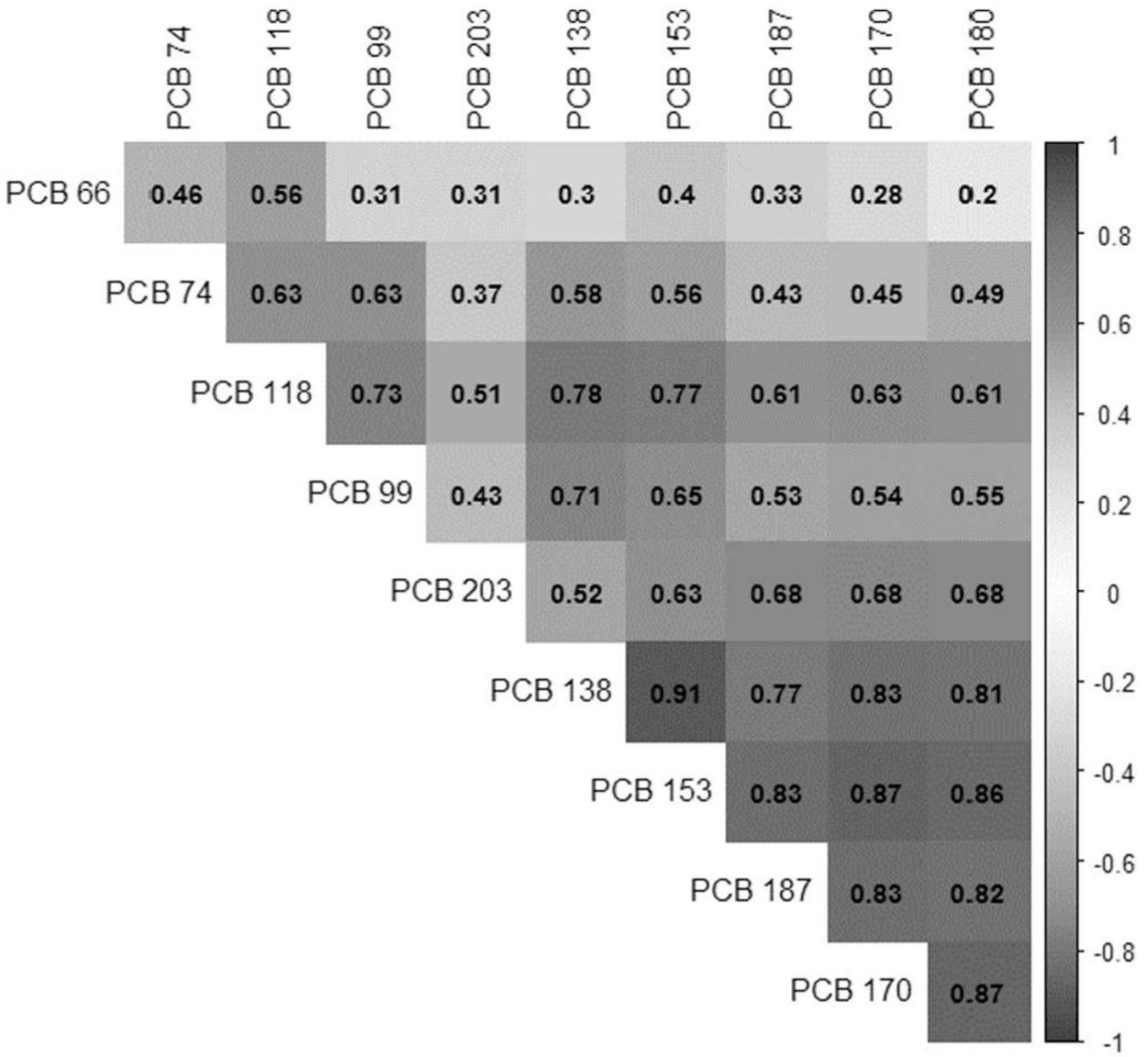
Heat map of correlations between PCB congeners.

To explore associations between disinhibitory behaviors and the combination of serum PCB congeners, we used weighted quantile sum (WQS) regression. Due to the high correlation of PCB congener exposures (see Fig. 1), this method was used to account for potential collinearity among the exposures (Carrico et al., 2015). In our analysis, quartile-scored PCB congeners within the generated PCB index were derived and empirically weighted for each individual based on bootstrap sampling (n = 100), and the weighted quantile score estimated the mean of all estimates. For binary outcomes, we incorporated the PBC index scores into multivariable logistic regression models adjusted for previously mentioned confounders. Likewise, for frequency of disinhibitory behavior outcomes, we incorporated the index into multivariable linear regression models. All statistical analyses were conducted with a 5% level of significance using SAS 9.4 statistical software.

## 3. Results

In our sample, 49.5% of the subjects were male, and the majority (59.6%) were over 16 years of age. A large portion of our study population was White (70.9%), followed by Black (19.9%), which is consistent with the 73.6% and 22.8% distribution, respectively, of this area according to the 1960 census. In terms of socioeconomic status, a greater percentage of fathers (29.7%) reported having a college degree than mothers (21.7%), and only 15.8% of subjects came from family with a household income greater than $10,000 per year. Sociodemographic characteristics of subjects and parents are detailed in Table 1.

In the univariable analysis of potential confounding factors (Table 2), we observed significantly higher PCB levels in mothers older than 35 years [Odds ratio (OR) = 3.17, 95% CI (1.11, 12.40), *P* = 0.03]. PCB levels were also 86% lower in mothers from households where the highest educated member graduated from high school compared to those from families with no high school graduates (OR = 0.14; 95% CI 0.02, 0.84, *P* = 0.03). Notably, PCB levels were over three times higher among mothers that consumed alcoholic beverages during pregnancy. However, there were no statistically significant results observed for PCB levels and either parent’s smoking status during pregnancy.

A comparison of categories of PCB levels between children reporting disinhibitory behaviors and those reporting no such behaviors is shown in Table 3. Interestingly, the adjusted odds ratio (aOR) for being a current smoker, but not for ever smoking, was higher in subjects within the third quartile of maternal PCB 66 exposure compared to those below the median (aOR = 1.93; 95% CI 1.05, 3.55). Although odds remained higher for those with the fourth quartile of PCB 66 exposure, these results were not statistically significant (aOR = 1.27; 95% CI 0.68, 2.35). The aOR for drinking over two alcoholic beverages per week, were higher for adolescents within the third (aOR = 1.46; 95% CI 0.86, 2.47) and fourth quartile of PCB 66 exposure (aOR = 1.39; 95% CI 0.83, 2.35), but the differences did not reach statistical significance. Odds of currently smoking, ever smoking one cigarette per day, and drinking over two alcoholic beverages per week were also higher for adolescents within the third and fourth quartile of PCB 118 exposure, though none of these results were statistically significant. There were no obvious patterns of association between disinhibitory behaviors and other PCB congeners or total PCB exposure. In our analysis of disinhibitory frequency and PCB exposures, there were no noticeable patterns of association for either PCB congener or total PCB exposure (Table 4).

**Fig. 2.**
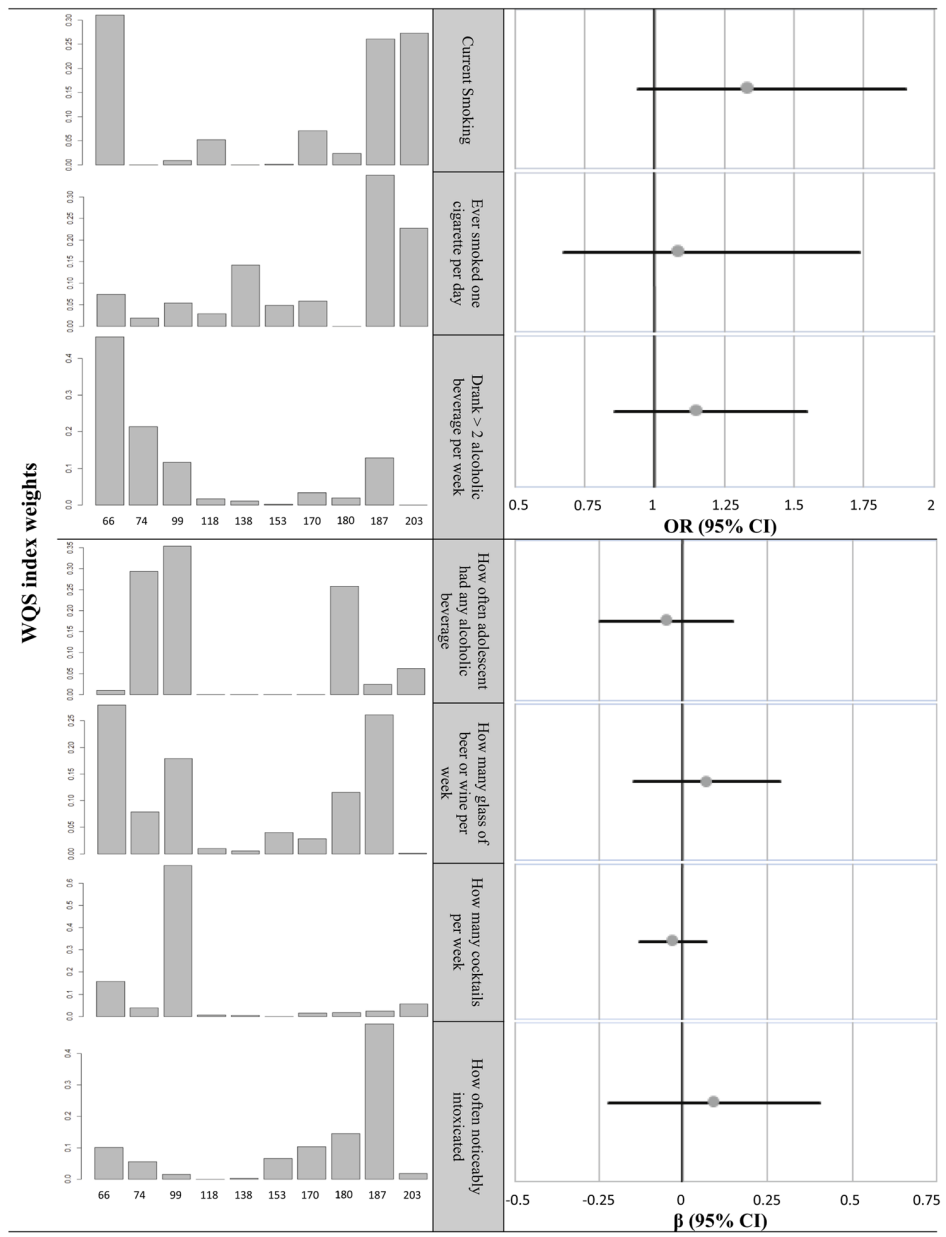
Association between PCB congener levels and disinhibitory behaviors based on weighted quantile sum (WQS) regression analysis. All models are adjusted for gender, mother’s race, mother’s age at time of birth highest parental education, and mother’s alcohol consumption during pregnancy, father preconception alcohol consumption, and who subjects drank with.

In our WQS analysis, we observed an 8 to 34% increase in odds of engaging in disinhibitory behavior for every unit increase in mixed PCB index, though none of these results were statistically significant (Fig. 2). For the analysis of PCB mixtures and current smoking status, the PCB congeners with the most weight were 66 (31%), 203 (27%) and 187 (26%). The analysis for smoking at least one cigarette per day also weighed heavily on congeners 187 (35%) and 203 (23%), and the greatest weight for drinking at least two alcoholic beverage per week was given to PCB 66 (46%). These results indicate that any potential association observed for odds of risk taking behavior may be driven by PCB congeners 66,187, and 203 in our results. For continuous measures of disinhibitory behaviors, there was no clear pattern of association between frequency of behaviors and PCB mixtures index. However, PCB congeners 66 and 187 again exhibited increased weighting in the positive, yet not significant, associations seen for the number of glasses of beer or wine consumed each week (PCB 66 = 28% and PCB 187 = 26%) and how often the subject was visibly intoxicated (PCB 187 = 50%).

## 4. Discussion

The onset of disinhibitory behaviors such as tobacco and alcohol use cluster in adolescence and shape adult behavior (DuRant et al., 1999; Kipping et al., 2012; Olsson et al., 2016). Animal studies demonstrate that in utero exposure to PCBs can disrupt neural circuitries, which potentially could enhance disinhibitory behaviors such as tobacco and alcohol use (Bell, 2014; Mariussen and Fonnum, 2001; Altmann et al., 2001). Yet, the potential effects of prenatal exposure to these environmental contaminants on adolescent substance use have, to our knowledge, never been studied in humans. Prior research on maternal predictors of disinhibitory behaviors have tended to conclude that genetic factors, rather than environmental factors are the primary predictors (King et al., 2009). Additionally, previous work examining environmental factors of disinhibitory behaviors in this CHDS cohort have focused on socioeconomic factors such as neighborhood poverty, but found no association with adolescent behaviors (Keyes et al., 2011). Our current study sought to add to the current body of knowledge on whether other environmental factors such as PCB exposure could play a key role in adolescent disinhibitory behavior.

The results from this prospective cohort study suggest that prenatal exposure to PCBs, including total PCB exposure, was not generally related to disinhibitory behaviors of smoking and alcohol use, among adolescents. However, our results revealed increased odds for being a current smoker in subjects prenatally exposed specifically to higher concentrations of PCB 66. The odds for drinking over two alcoholic beverages per week were also higher for adolescents highly exposed to PCB 66 in utero, but the differences did not reach statistical significance. These results may indicate that this PCB congener could potentially play a role in multifactorial inducement of neurodevelop-mental alterations that increase the risk of becoming a long-term user of tobacco and possibly alcohol. This is in line with a previous retrospective cohort study that found early-life exposure to the solvent tet-rachloroethylene via contaminated drinking water to be associated with increased risk of using two or more illicit drugs as teenager or adults with 50-60% (Aschengrau et al., 2011).

Health effects linked to PCBs are the subject of numerous research studies. Evidence show that this group of chemicals can disrupt the endocrine system, and exhibit a broad range of adverse effects in humans and other species. It is also clear that the individual PCB congeners have different toxicological profiles and induce diverse biological responses in experimental models as well as in humans. For example, certain PCBs produce a dioxin-like response through steric homology with 2,3,7,8-tetrachlorodibenzodioxin (TCDD) and interactions with the aryl hydrocarbon receptor (AhR). Other congeners are neurotoxic, mimic endogenous hormone action, or interfere with their metabolism in specific ways (Wolff et al., 1997; Warner et al., 2012). In utero exposure to different PCB congeners can also induce diverse, and even opposite, effects in brain regions important for decision-making and pleasure-seeking behaviors (Seegal et al., 2005). PCB 66 lacks one out of four geometric and substituent positions descriptors to be fully referred to as dioxin-like (Van den Berg et al., 1998), but can be classified as potentially antiestrogenic and may thereby affect the developing brain (Wolff et al., 1997; Warner et al., 2012; McCarthy, 2008). Our findings illustrate that it is warranted to further investigate mechanisms by which PCB 66 possibly may disrupt neurodevelopment.

We compared the non-ortho and mono-ortho substituted congeners PCB 66, 74 and 188 that belong to group 2A in the Wolff classification system (Wolff et al., 1997; Warner et al., 2012). Although the correlations between the dioxin-like PCB 118, and the potentially dioxin-like congeners PCB 66 and 74 are overall moderate (r = 0.46 to 0.63), results for associations between disinhibitory behaviors and high levels of PCB 74 were generally negative, while results for PCB 118 were positive, yet neither reached significance. Notably, there was a consistent bell-shape seen for many of our exposure-behavior relationships, with some PCBs exhibiting negative-positive-negative association between quartiles, and others exhibiting positive-negative-positive associations between quartiles. This threshold dose-response relationship is commonly observed for toxicants available at persistent and low concentrations (Lagarde et al., 2015). Additionally, because EDCs, like PCBs, impact regulation of several different biological pathways and processes, this bell-shape is commonly reported for epidemiological studies of EDCs (Lagarde et al., 2015). Therefore, assessing risk of disinhibitory behaviors in relation to PCB exposures will require further study of the biological mechanisms of these associations.

Maternal PCB exposure can occur through a variety of sources, including dietary intake of fish or meat, and the compounds accumulate in lipid rich tissues (DeKoning and Karmaus, 2000). As fat is rapidly broken down during the third trimester of pregnancy and during lactation, the lipophilic PCBs can be released and transferred across the placenta to the fetus or to infants via breastmilk (Herrera and Ortega- Senovilla, 2014; Herrera, 2002). Our results showed that the total PCB levels were over three times higher for mothers that consumed alcoholic beverages during pregnancy than those who did not drink alcohol. We find these results striking and noteworthy for future research. Specifically, research has documented alcohol consumption during pregnancy to be a primary cause of fetal alcohol syndrome (Gupta et al., 1996), and public health prevention campaigns have promoted abstinence of alcohol during pregnancy. While our study was not powered to test the interaction between PCB exposure and maternal alcohol consumption on disinhibitory behavior, these findings could potentially add further evidence for potential deleterious effects on child and adolescent outcomes. There may also be several plausible biological explanations for the increased PCB levels, including effects of alcohol on PCB uptake, metabolism and accumulation.

In our study, we found no sex-specific effects although sex differences could have been expected based on previous work conducted on rats suggested that estrogen was a mediating factor between development PCB exposure and alcohol consumption (Lombardo and Peck, 2018).

Despite the strengths of using prospectively collected data to measure PCB exposures prior to exhibited behaviors in adolescence, there were some limitations of our study. First, we conducted several statistical tests in our analysis, and thus, we cannot rule out the potential that some of the significant results seen may be chance findings. However, considering our conservative sample size, the attenuating effect of our included confounders, and the fact that only a few of our results were significant, this risk is minimal. Second, we did not have information on breastfeeding, which may be a primary source of PCBs in infancy. The specific purpose of this study was nevertheless to evaluate associations with prenatal PCB exposures on later adolescent behaviors. Moreover, during the 1960s, breastfeeding practices were highly influenced by education and income level of mothers (Lactation IoMUCiNSDPa, 1991). Therefore, by adjusting for parental education in our analysis, we may have indirectly adjusted for breastfeeding. Likewise, self-report of maternal smoking during pregnancy may have resulted in underreporting. However, it is important to note that data on maternal smoking during pregnancy was gathered in the 1960s when 33.9% of women were smokers, and there was less knowledge about the consequences of such behaviors (CDC CfDCaP, 2002). In addition, studies have shown associations between smoking in women and educational attainment (CDC CfDCaP, 2002). Thus, by adjusting for these factors in our analysis we may have also improved robustness of our findings through properly specifying the model. Third, we used postpartum serum samples drawn within three days of delivery to estimate in utero PCB exposures. Nevertheless, due to the long half-life of PCBs, previous studies have established postpartum PCB levels to be a valid measure of levels across pregnancy (Longnecker et al., 1999). Fourth, adolescent behaviors were based on self-report of the children, and measurement error is inevitable in any study of unfavorable behaviors such as those reported here. However, we have no reason to believe that differences in reporting would vary by PCB exposure level, but any potential mis-classification would be expected to diminish the magnitude of our effect sizes. Additionally, this study began in the 1960s, with study subjects reaching adolescence during the 1970s. Trends in alcohol, cigarette, and other substance use have changed across time, with a drastic reduction for both adolescents and pregnant mothers (Johnson and Gerstein, 1998). PCBs were also banned in the US in 1977, and exposures to the general population decreased shortly after that (Hopf et al., 2009). However, due to their persistence and the previous widespread use PCB levels in the general population continue to be of concern.

## 5. Conclusion

This prospective epidemiological study, adds new evidence to a long-standing debate about whether genetic or environmental and parental behavioral factors during pregnancy are the primary predictors of behavioral disinhibition in adolescence, specifically alcohol use and smoking. In particular, we added evidence that early-life exposures to environmental contaminants may evoke adverse behavior later in life. The results revealed increased odds for being a current smoker in subjects prenatally exposed to higher concentrations of PCB 66, but none of the other measured congeners. It is known that the different PCB congeners induce diverse biological responses. However, the prenatal PCB exposure—offspring substance use association is insufficiently investigated, and potential causal mechanisms are not well understood. We recommend further research on this topic, which not only includes replicating this study in different and larger longitudinal samples, but also detailed animal experimental studies of potential underlying mechanisms are warranted. In addition, mediating relationships of psychosocial stressors including adverse childhood events and other exogenous environmental exposures such as neighborhood stress, should be examined. Identification of potential risk factors could influence success of interventions for particularly vulnerable populations.

## Figures and Tables

**Table 1:** Descriptive statistics for study subjects (*n* = 554).

		N	%
Male		274	49.46
Age (years)	15–16	224	40.43
	17–18	330	59.57
Mother’s race	White	393	70.94
	Black	110	19.86
	Hispanic	18	3.25
	Asian	32	5.78
	Other	1	0.18
Mother’s age at child’s birth (years)	15–24	152	27.44
	25–34	293	52.89
	35–47	109	19.68
Mother’s education^a^	Less than high school	64	11.57
	High school graduate	180	32.55
	Trade school	35	6.33
	Some college	154	27.85
	College graduate	120	21.70
Father’s education^b^	Less than high school	81	14.67
	High school graduate	167	30.25
	Trade school	20	3.62
	Some college	120	21.74
	College graduate	164	29.71
Household income^c^ (USD per year)	< $5000	114	22.80
	$5000 to $10,000	307	61.40
	> $10,000	79	15.80
Smoking cigarettes		94	16.97
Alcohol consumption	Beer^d^	279	53.14
	Wine^e^	215	41.35
	Mixed cocktails^f^	233	44.81

a Mother’s education level is missing for 1 subject.

b Father’s education level is missing for 2 subject.

c Household income missing for 54 subjects.

d Beer consumption missing for 29 subjects.

e Wine consumption missing for 34 subjects.

f Mixed cocktails consumption missing for 34 subjects.

**Table 2:** Association between total PCB concentrations and potential confounding factors using.

n = 554		N (%)	OR	95% CI	*P*-value
Mother’s age at child’s birth	15–24	152 (27.4)	REF	-	-
	25–34	293 (52.9)	2.40	(0.83, 6.92)	0.10
	35–47	109 (19.7)	**3.71**	**(1.11, 12.40)**	**0.03**
Household income (per year)	< $5000	114 (22.8)	REF	-	-
	$5000 to $10,000	307 (61.4)	1.44	(0.47, 4.45)	0.53
	> $10,000	79 (15.8)	1.78	(0.43, 7.45)	0.43
Parental education level^a^	< High school	30 (5.4)	REF	-	-
	High school graduate	141 (25.5)	**0.14**	**(0.02, 0.84)**	**0.03**
	Trade school	31 (5.6)	0.22	(0.02, 2.78)	0.24
	Some college	151 (27.3)	0.42	(0.42, 2.24)	0.31
	College graduate	201 (36.3)	1.08	(0.23, 5.09)	0.92
Mother’s alcohol consumption during pregnancy (≥ once per week)^b^	Beer	69 (16.1)	**3.23**	**(1.21, 8.64)**	**0.02**
	Wine	84 (19.5)	**3.06**	**(1.18, 7.91)**	**0.02**
	Whisky	81 (18.8)	**3.25**	**(1.25, 8.47)**	**0.02**
Father’s alcohol consumption prior to pregnancy (≥ once per week)^c^	Beer	236 (56.7)	1.49	(0.61, 3.65)	0.38
	Wine	145 (35.0)	**2.55**	**(1.03, 6.27)**	**0.04**
	Whisky	220 (53.9)	**4.00**	**(1.41, 11.43)**	< **0.01**
Mother’s cigarette smoking during pregnancy^d^		165 (30.2)	0.78	(0.29, 2.11)	0.63
Father’s cigarette smoking during pregnancy^e^		281 (52.0)	0.96	(0.39, 2.37)	0.92
Who subjects drank with^f^	Did not drink	127 (23.6)	REF	-	-
	Alone	6 (1.1)	0.50	(0.01, 46.63)	0.76
	With parents	46 (8.6)	0.81	(0.14, 4.56)	0.80
	With a friend	83 (15.4)	0.28	(0.58, 1.36)	0.12
	In a group or at a party	143 (26.6)	0.58	(0.17, 2.03)	0.40
	Any combination of options	133 (24.7)	0.50	(0.14, 1.84)	0.30

a Parental education is based on the highest education level of the mother or father.

b Maternal alcohol consumption during pregnancy missing for 124 subjects.

c Paternal alcohol consumption prior to conception missing for 136 subjects.

d Maternal smoking during pregnancy missing for 8 subjects.

e Paternal smoking during pregnancy missing for 14 subjects.

f Who adolescents drank with missing for 16 subjects.

**Table 3:** Odds of disinhibitory behavior for children and PCB exposures, based on logistic regression.

Lipid-adjusted PCB congener	Below median yes/no	Third quartile aOR (95% CI)		Forth quartile aOR (95% CI)	
		
	Referent group	Yes/no	aOR (95% CI)	Yes/no	aOR (95% CI)
Current smoking^a^					
PCB 66	44/230	28/110	1.93 (1.05, 3.55)	22/120	1.27 (0.68, 2.35)
PCB 74	58/218	22/115	0.79 (0.44, 1.41)	14/127	0.53 (0.27, 1.05)
PCB 99	47/228	24/113	1.25 (0.69, 2.27)	22/119	1.23 (0.67, 2.26)
PCB 118	48/229	25/114	1.47 (0.81, 2.64)	21/117	1.11 (0.59, 2.08)
PCB 138	51/24	26/110	1.15 (0.63, 2.09)	17/126	0.74 (0.38, 1.42)
PCB 153	54/221	21/117	0.76 (0.42, 1.41)	19/122	0.87 (0.41, 2.00)
PCB 170	48/228	25/112	1.24 (0.68, 2.26)	21/120	0.94 (0.50, 1.76)
PCB 180	53/223	21/117	0.88 (0.48, 1.63)	20/120	0.90 (0.48, 1.68)
PCB 187	49/227	25/113	1.00 (0.55, 1.80)	20/120	1.01 (0.53, 1.94)
PCB 203	46/230	29/109	1.78 (0.98, 3.23)	19/121	0.86 (0.45, 1.65)
ƩPCB	53/223	22/116	0.87 (0.48, 1.58)	19/121	0.88 (0.46, 1.58)
Ever smoked one cigarette per day^a,b^					
PCB 66	23/209	9/103	0.85 (0.36, 1.99)	11/111	0.98 (0.44, 2.20)
PCB 74	21/197	12/107	1.03 (0.47, 2.25)	10/119	0.90 (0.38, 2.09)
PCB 99	24/206	8/103	0.73 (0.30, 1.76)	11/114	1.16 (0.52, 2.60)
PCB 118	22/205	11/105	1.11 (0.49, 2.48)	10/113	1.17 (0.50, 2.74)
PCB 138	20/203	12/101	1.37 (0.60, 3.12)	11/119	1.45 (0.61, 3.45)
PCB 153	18/204	15/103	2.07 (0.95, 4.50)	10/116	1.30 (0.53, 3.17)
PCB 170	21/207	14/101	1.64 (0.74, 3.64)	8/115	0.83 (0.34, 2.09)
PCB 180	21/201	14/107	1.30 (0.59, 2.84)	8/115	0.76 (0.31, 1.90)
PCB 187	22/205	10/102	1.01 (0.44, 2.34)	11/116	1.27 (0.53, 3.01)
PCB 203	20/209	9/102	1.13 (0.46, 2.77)	14/112	1.48 (0.67, 3.32)
ƩPCB	22/200	11/107	1.10 (0.49, 2.47)	10/116	1.12 (0.47, 2.69)
Drank > 2 alcoholic beverage per week^a^					
PCB 66	97/177	52/86	1.46 (0.86, 2.47)	53/89	1.39 (0.83, 2.35)
PCB 74	102/174	55/82	1.07 (0.65, 1.77)	44/96	0.98 (0.58, 1.67)
PCB 99	101/174	52/85	1.05 (0.63, 1.75)	49/93	0.98 (0.59, 1.64)
PCB 118	99/178	53/86	1.07 (0.64, 1.79)	50/88	1.15 (0.68, 1.96)
PCB 138	100/175	59/79	1.17 (0.69, 1.97)	43/98	0.93 (0.54, 1.58)
PCB 153	101/178	59/79	1.42 (0.85, 2.39)	42/95	0.91 (0.53, 1.56)
PCB 170	107/169	54/83	1.13 (0.67, 1.90)	41/100	0.66 (0.39, 1.11)
PCB 180	101/175	57/81	1.05 (0.63, 1.77)	44/96	0.77 (0.45, 1.31)
PCB 187	109/167	52/86	0.91 (0.55, 1.51)	41/99	0.72 (0.41, 1.25)
PCB 203	108/168	48/90	0.75 (0.45, 1.38)	46/94	0.73 (0.40, 1.35)
ƩPCB	105/171	55/83	1.07 (0.65, 1.78)	42/98	0.73 (0.43, 1.25)

a Model adjusted for gender, mother’s race, mother’s age at time of birth, highest parental education, mother’s alcohol consumption during pregnancy, father’s preconception alcohol consumption, and who subjects drank with.

b Ever smoking 1 cigarette per day is missing for 88 subjects.

**Table 4:** Associations between disinhibitory behavior frequency and PCB exposures, based on negative binomial models.

Lipid-adjusted PCB congener	Second quartile^a^ aRR (95% CI)	Third quartile^a^ aRR (95% CI)	Forth quartile^a^ aRR (95% CI)
How often adolescent had any alcoholic beverage			
PCB 66	0.96 (0.83, 1.12)	1.01 (0.87, 1.17)	0.76 (0.84, 1.13)
PCB 74	0.98 (0.85, 1.14)	1.00 (0.87, 1.16)	0.97 (0.84, 1.13)
PCB 99	0.95 (0.82, 1.10)	1.00 (0.86, 1.16)	0.98 (0.84, 1.14)
PCB 118	0.88 (0.76, 1.03)	0.95 (0.82, 1.09)	0.93 (0.80, 1.08)
PCB 138	1.00 (0.87, 1.16)	0.99 (0.85, 1.15)	0.95 (0.82, 1.11)
PCB 153	0.96 (0.83, 1.11)	1.01 (0.88, 1.18)	0.92 (0.78, 1.07)
PCB 170	0.98 (0.85, 1.13)	0.94 (0.81, 1.09)	0.89 (0.77, 1.04)
PCB 180	1.08 (0.93, 1.25)	0.97 (0.84, 1.12)	0.96 (0.82, 1.12)
PCB 187	1.07 (0.93, 1.24)	0.99 (0.85, 1.15)	0.91 (0.77, 1.07)
PCB 203	0.94 (0.81, 1.09)	0.97 (0.83, 1.13)	0.95 (0.82, 1.10)
ƩPCB	1.03 (0.89, 1.19)	0.98 (0.85, 1.14)	0.94 (0.81, 1.10)
How many glass of beer or wine per week			
PCB 66	0.88 (0.71, 1.09)	1.18 (0.96, 1.45)	1.03 (0.84, 1.26)
PCB 74	0.90 (0.73, 1.10)	0.96 (0.79, 1.17)	0.87 (0.70, 1.07)
PCB 99	0.93 (0.76, 1.14)	0.85 (0.69, 1.05)	0.95 (0.77, 1.16)
PCB 118	0.85 (0.69, 1.05)	0.96 (0.79, 1.17)	0.91 (0.74, 1.12)
PCB 138	0.86 (0.70, 1.05)	1.03 (0.84, 1.26)	0.90 (0.73, 1.12)
PCB 153	0.90 (0.74, 1.11)	1.02 (0.84, 1.23)	0.92 (0.74, 1.14)
PCB 170	0.94 (0.76, 1.15)	1.00 (0.81, 1.22)	0.95 (0.77, 1.17)
PCB 180	1.04 (0.85, 1.29)	0.96 (0.78, 1.18)	1.02 (0.82, 1.25)
PCB 187	1.14 (0.93, 1.40)	1.08 (0.88, 1.33)	1.01 (0.81, 1.26)
PCB 203	0.88 (0.72, 1.09)	0.95 (0.77, 1.17)	0.94 (0.76, 1.16)
ƩPCB	0.91 (0.74, 1.12)	0.98 (0.80, 1.19)	0.92 (0.74, 1.13)
How many cocktails per week^b^			
PCB 66	0.89 (0.65, 1.23)	1.03 (0.75, 1.40)	1.02 (0.76, 1.38)
PCB 74	0.95 (0.70, 1.29)	0.98 (0.72, 1.33)	1.03 (0.75, 1.42)
PCB 99	0.83 (0.60, 1.14)	1.05 (0.77, 1.43)	1.12 (0.82, 1.53)
PCB 118	0.80 (0.57, 1.10)	1.10 (0.81, 1.49)	1.06 (0.78, 1.45)
PCB 138	1.03 (0.76, 1.39)	0.98 (0.72, 1.35)	1.12 (0.82, 1.53)
PCB 153	1.01 (0.75, 1.36)	1.10 (0.82, 1.49)	0.89 (0.64, 1.24)
PCB 170	0.99 (0.73, 1.35)	1.15 (0.85, 1.54)	0.86 (0.63, 1.19)
PCB 180	0.97 (0.73, 1.32)	0.93 (0.69, 1.26)	0.86 (0.62, 1.18)
PCB 187	1.23 (0.90, 1.65)	1.15 (0.84, 1.56)	0.93 (0.66, 1.31)
PCB 203	0.91 (0.66, 1.24)	1.05 (0.77, 1.42)	0.93 (0.68, 1.28)
ƩPCB	1.07 (0.79, 1.45)	1.09 (0.80, 1.47)	1.05 (0.75, 1.45)
How often noticeable intoxicated			
PCB 66	1.10 (0.89, 1.35)	0.94 (0.76, 1.16)	1.14 (0.93, 1.40)
PCB 74	1.01 (0.83, 1.24)	1.17 (0.95, 1.42)	1.19 (0.97, 1.46)
PCB 99	0.95 (0.78, 1.17)	0.96 (0.78, 1.18)	1.01 (0.82, 1.25)
PCB 118	0.99 (0.81, 1.22)	0.97 (0.79, 1.20)	0.94 (0.76, 1.16)
PCB 138	0.93 (0.76, 1.14)	0.92 (0.75, 1.14)	1.08 (0.88, 1.33)
PCB 153	0.86 (0.69, 1.05)	0.88 (0.71, 1.08)	1.11 (0.90, 1.36)
PCB 170	0.96 (0.78, 1.17)	0.93 (0.75, 1.14)	1.17 (0.96, 1.43)
PCB 180	0.91 (0.74, 1.13)	1.06 (0.86, 1.29)	1.12 (0.91, 1.38)
PCB 187	0.98 (0.80, 1.20)	1.16 (0.94, 1.42)	1.07 (0.86, 1.32)
PCB 203	0.99 (0.81, 1.23)	0.88 (0.72, 1.09)	1.05 (0.85, 1.29)
ƩPCB	0.93 (0.76, 1.14)	0.98 (0.80, 1.20)	1.13 (0.92, 1.40)

a Model adjusted for gender, mother’s race, mother’s age at time of birth, highest parental education, and mother’s alcohol consumption during pregnancy, father preconception alcohol consumption, and who subjects drank with.

b This model is also zero-inflated.
